# Characterization of Critically Ill COVID-19 Patients at a Brooklyn Safety-Net Hospital

**DOI:** 10.7759/cureus.9809

**Published:** 2020-08-17

**Authors:** Stephen Capone, Shogik Abramyan, Brent Ross, Joshua Rosenberg, John Zeibeq, Viswanath Vasudevan, Reza Samad, Louis Gerolemou, Evgeny Pinelis, James Gasperino, Jose Orsini

**Affiliations:** 1 Medicine, St. George's University School of Medicine, St. George, GRD; 2 Department of Surgery, The Brooklyn Hospital Center, Academic Affiliate of the Icahn School of Medicine at Mount Sinai, Clinical Affiliate of the Mount Sinai Hospital, Brooklyn, USA; 3 Division of Critical Care Medicine, The Brooklyn Hospital Center, Academic Affiliate of the Icahn School of Medicine at Mount Sinai, Clinical Affiliate of the Mount Sinai Hospital, Brooklyn, USA

**Keywords:** coronavirus, sars-cov-2 (severe acute respiratory syndrome coronavirus -2), covid-19, intensive care, critical care

## Abstract

Background

The novel coronavirus disease 2019 (COVID-19) pandemic continues to spread across the country with over 3 million cases and 150,000 deaths in the United States as of July 2020. Outcomes have been poor, with reported admission rates to the intensive care team of 5% in China and mortality among critically ill patients of 50% in Seattle. Here we explore the disease characteristics in a Brooklyn safety-net hospital affected by the severe acute respiratory syndrome coronavirus 2 (SARS-CoV-2) pandemic.

Methods

A retrospective chart review of COVID-19 positive patients at The Brooklyn Hospital Center who were treated by the intensive care team prior to April 20, 2020. Data was extracted from the electronic health record, analyzed and correlated for outcome.

Results

Impact of various clinical treatments was assessed, showing no change in median overall survival (OS) of both hydroxychloroquine with azithromycin or vitamin C with zinc. Supplemental therapies were used in selected patients, and some were shown to increase median OS and patients requiring vasopressor support or invasive mechanical ventilation showed decreased OS. There was no statistically significant difference in overall survival based on ethnicity, healthcare status, or individual medical comorbidities, although a negative trend exists for diabetes. Despite this, there is a trend towards increasingly poor prognosis based on the number of comorbidities and Class 3 obesity.

Conclusions

Despite the fact that we show no significant differences in mortality based on ethnicity, insurance status, or individual medical comorbidities, we show a high overall mortality. There is also a trend towards increased overall mortality in Class 3 obesity, which should be further investigated. We suggest that these findings may be attributed to both socioeconomic factors and an increased incidence of total medical comorbidities in our patient population.

## Introduction

The novel coronavirus disease 2019 (COVID-19) pandemic continues to spread across the country with over 3 million cases and 150,000 deaths nationwide as of July 2020. Outcomes have been poor despite our best efforts, with reported admission rates to the intensive care unit upwards of 15% in China and Italy, and mortality among these patients as high as 50% in Seattle [1-4]. In New York City (NYC), the epicenter of this outbreak, patients showed an overall mortality of 10.2% and those requiring mechanical ventilation, approximately one-third of critically ill patients, had mortality between 14.6 and 24.5% [5,6]. A recent report noted an overall mortality of critical care patients in Manhattan of almost 40% [7]. The Brooklyn Hospital Center is a 464-bed community safety-net hospital in Brooklyn, NY. The purpose of this study was to explore the disease characteristics in a Brooklyn safety-net hospital affected by the severe acute respiratory syndrome coronavirus 2 (SARS-CoV-2) pandemic and the clinical course and outcomes of this uniquely diverse population. 

## Materials and methods

Study population and data sources

This study was performed at The Brooklyn Hospital Center and approved by the institutional review board (#1595421). A retrospective review of the electronic medical record (EMR; Allscripts Healthcare Solutions) was utilized to identify all patients at our institution with confirmed SARS-CoV-2 infections (n=1084). All tests were performed by nasopharyngeal or sputum samples and qualitative detection of nucleic acid performed by reverse transcriptase polymerase chain reaction (RT-PCR; Abbott, Chicago, IL). We then identified each individual patient admitted to the hospital and managed by the intensive care team (n=102). This included patients in the medical intensive care unit (MICU), surgical intensive care unit (SICU), cardiac care unit (CCU), cardiac telemetry unit (CSCU), cardiac progressive care unit (CPCU), and post-anesthesia care unit (PACU). Clinical outcomes were assessed until our study cutoff date of April 20, 2020. Pre-existing medical comorbidities were identified in the EMR and noted. All demographic and clinical data were manually extracted from the individual EMR by physicians. Even if patients were not offered a mechanical ventilator due to do-not-resuscitate (DNR) status, they were included in the study.

Statistical analysis

All statistical analyses were performed using Prism v. 8.4.2 (GraphPad Software, LLC, San Diego, CA). Statistical significance was defined as p<0.05. Descriptive statistics were reported as means with interquartile ranges, as relevant. Categorical variables were reported as total number and percentages. No imputation was made for missing data. Characteristics of each group were compared by analysis of variance (ANOVA). Survival curve analyses were performed using log-rank (Mantel-Cox) tests. Correlation tests were utilized to calculate Pearson correlation coefficient (Pearson r) values and multiple linear regressions utilized least squares. Days to death was calculated from the date of admission and patients who were discharged from the hospital were censored at that time, though it should be noted that patients arrived at differing stages of COVID-19, with some being directly admitted to the intensive care service while others admitted to the inpatient unit prior to upgrade to this service.

## Results

**Figure 1:**
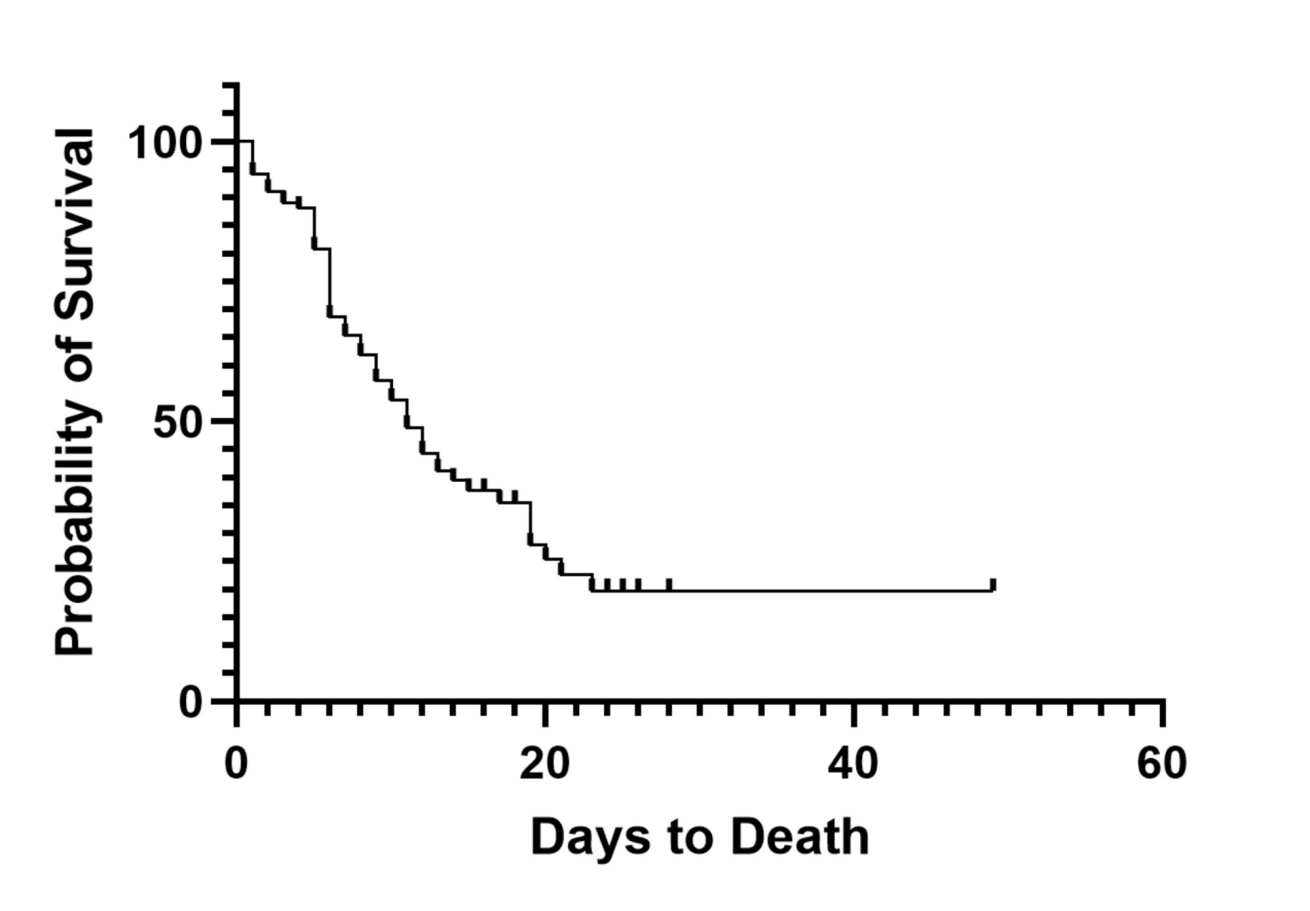
Overall Survival of Critically Ill Patients

**Figure 2:**
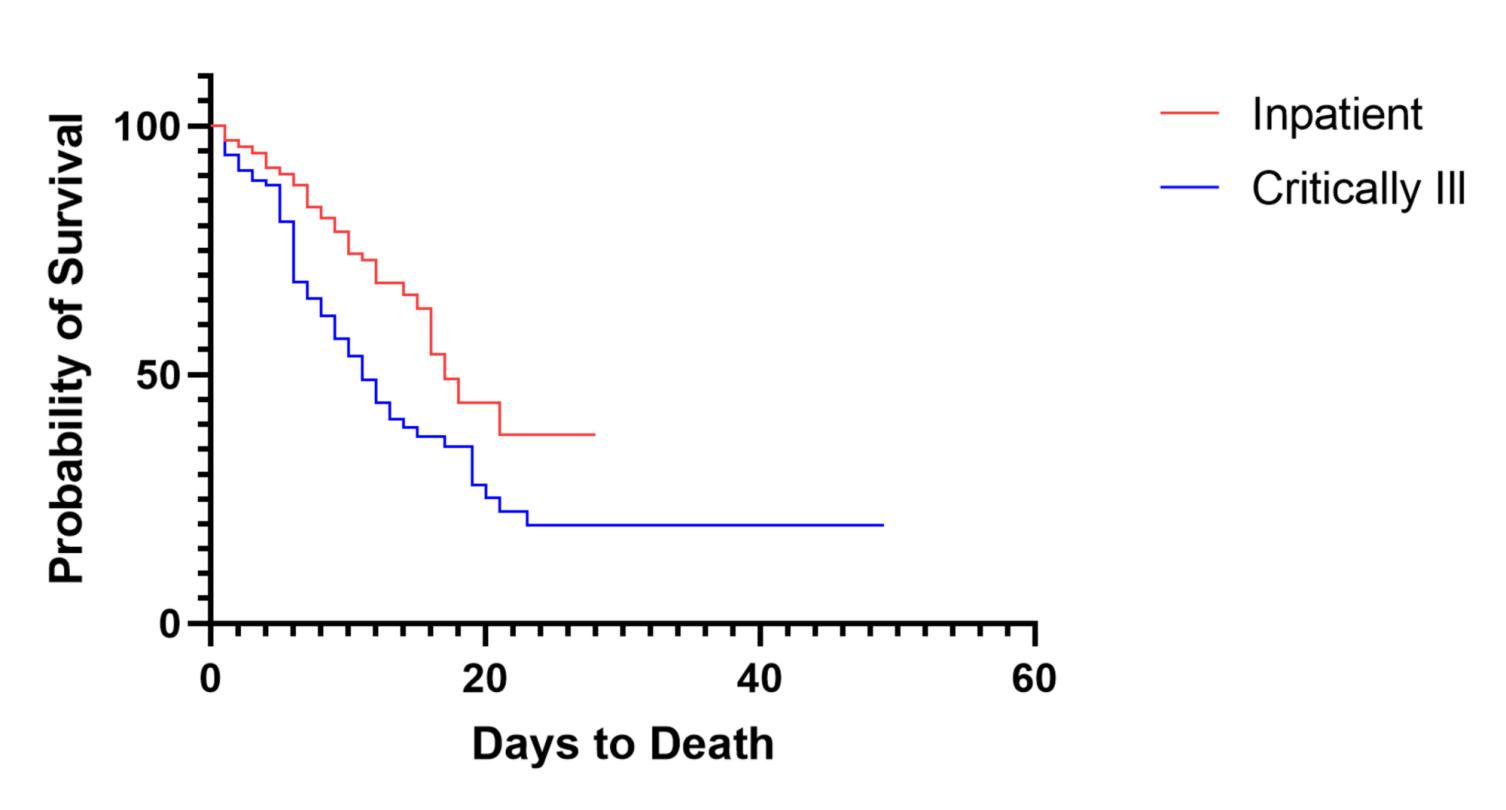
Overall Survival of Inpatient COVID-19 Patients Versus Critically Ill COVID-19 Patients

During the study period from March 1, 2020 to April 20, 2020, we tested 2003 patients, with 1084 positive results and 29 pending, of which we identified 493 admitted to the hospital. Of the admitted patients, 102 (20.7%) were admitted to the intensive care service prior to April 20, 2020, with 31 still currently admitted (30.4%) at the end of the study period. The median age of the cohort was 63 years and 82.4% were African American or Hispanic (Table 1). Median length of stay (LOS) for all critically ill patients was 11 days from hospital admission, and after controlling for DNR status the overall all-cause mortality was 53.6% (37/69, Figure 1). Median hospital overall survival (OS) for inpatient COVID-19 patients was significantly longer than intensive care patients (17 days vs. 11 days, p<.001, Figure 2). Preliminary evidence has suggested that minority groups have been disproportionately affected by COVID-19, with a higher percentage of minority patients occupying the intensive care unit than the general population with a higher overall mortality [5,8-10]. Amongst those admitted to the ICU, we observed no statistically significant difference in mortality among different ethnic groups (Table 1). During the study period, 94.1% (96/102) of patients received hydroxychloroquine in combination with azithromycin, and 71.6% (73/102) received supplementation with vitamin C and zinc, 40.2% (41/102) received anticoagulation, 21.6% (22/102) received tocilizumab, 20.6% received lopinavir/ritonavir (21/102), 36.3% (37/102) received oseltamivir, 1.0% (1/102) patients received remdesivir, and 82.4% (84/102) required vasopressor support. During the study period, a total of 10 patients were discharged from the intensive care service, with one direct discharge from the ICU home and nine transferred to the floor, with an average time to discharge of 14 days (interquartile range [IQR] 10.5-19.5 days). For the 61 patients who are deceased, the average time to death was 8.33 days (IQR 5-11 days).

**Table 1:** Characteristics of Critically Ill COVID-19 Patients * Vascular events included prior cerebrovascular accident, coronary artery disease, myocardial infarction, pulmonary embolism, deep vein thrombosis, peripheral arterial disease, HbSC, sickle cell disease †Obesity was defined as having a BMI ≥30

Characteristic	All Patients (N=102)	African American (N=52)	Hispanic (N=32)	Caucasian (N=7)
Demographics				
Median Age – yrs (IQR)	63.22 (53.3-74.3)	63.60 (57.0-76.0)	62.03 (49.8-73.5)	67.86 (64.0-74.0)
Male – no. (%)	55 (53.9)	25 (48.1)	19 (59.4)	5 (71.4)
Medicare/Medicaid – no. (%)	83 (81.4)	41 (78.8)	27 (84.4)	6 (85.7)
Self Pay – no. (%)	3 (2.9)	2 (3.8)	1 (3.1)	0 (0)
Private Insurance – no. (%)	16 (15.7)	9 (17.3)	4 (12.5)	1 (14.3)
Medical History				
Charlson Comorbidity Index – avg. (range)	3.38 (0-11)	3.35 (0-9)	3.44 (0-11)	3.57 (0-5)
Body Mass Index – avg.	30.98	32.68	29.87	27.89
Obesity† - no. (%)	44 (43.1)	26 (50.0)	13 (40.6)	2 (28.6)
Diabetes – no. (%)	50 (49.0)	27 (51.9)	16 (50.0)	3 (42.9)
Hypertension – no. (%)	61 (59.8)	30 (57.7)	20 (62.5)	5 (71.4)
Asthma – no. (%)	12 (11.8)	5 (9.6)	2 (6.3)	1 (14.2)
Vascular* – no. (%)	30 (29.4)	16 (30.8)	10 (31.3)	2 (28.6)
Chronic Kidney Disease – no. (%)	11 (10.8)	6 (11.5)	4 (12.5)	0 (0)
Gastroesophageal Reflux Disease (GERD) – no. (%)	10 (9.8)	5 (9.6)	3 (9.4)	2 (28.6)
Human Immunodeficiency Virus – no. (%)	5 (4.9)	4 (7.7)	1 (3.1)	0 (0)
Clinical Characteristics				
Hydroxychloroquine + Azithromycin – no. (%)	96 (94.1)	49 (94.2)	29 (90.6)	7 (100)
Vitamin C + Zinc – no. (%)	73 (71.6)	36 (69.2)	24 (75.0)	5 (71.4)
IVIG – no. (%)	8 (7.8)	0 (0)	6 (18.8)	1 (14.3)
Vasopressors – no. (%)	84 (82.4)	42 (80.8)	27 (84.4)	6 (85.7)
Anticoagulation – no. (%)	41 (40.2)	18 (34.6)	17 (53.1)	3 (42.9)
Invasive Mechanical Ventilation – no. (%)	90 (88.2)	44 (84.6)	30 (93.8)	6 (85.7)
Death – no. (%)	61 (59.8)	33 (63.5)	18 (56.3)	4 (57.1)
Discharge from Hospital – no. (%)	10 (9.8)	7 (13.5)	3 (9.4)	0 (0)

The average age of the cohort was 63.2 years, 53.9% were male, and a vast majority of patients were on Medicare or Medicaid (81.4%). Complete demographic, social, and clinical data separated by ethnicity is summarized in Table 1. Patients were divided into demographic groups as follows: African American (n=52), Hispanic (n=32), Caucasian (n=7) and other (n=11). Statistical analysis showed no difference in baseline clinical characteristics between these groups, including age (p=0.66), gender (p=0.38), or body mass index (BMI, p=0.34). We also found an overall average body mass index (BMI) of 30.98, which is categorized as obesity by the Centers for Disease Control and Prevention (CDC) [11]. 

Clinical course

A combination of hydroxychloroquine and azithromycin was used on 96 patients (96/102, 94.1%). Standard dosage of hydroxycholoroquine/azithromycin was 400mg Q12 for three doses, then daily/500mg QD, respectively. A combination of vitamin C and zinc was also utilized in 73 patients (71.6%) and supplemental intravenous immunoglobin in eight patients (7.8%). Vasopressor support was required in 84 of these critically ill patients (82.4%). Approximately 40% of patients were treated empirically with therapeutic anticoagulation for suspected pulmonary embolism (PE) or thrombotic microangiopathy based on provider judgement. Invasive mechanical ventilation was required in 90 (88.2%) of patients admitted to the ICU. Breakdown of these treatments by ethnicity are summarized in Table 1. 

**Figure 3:**
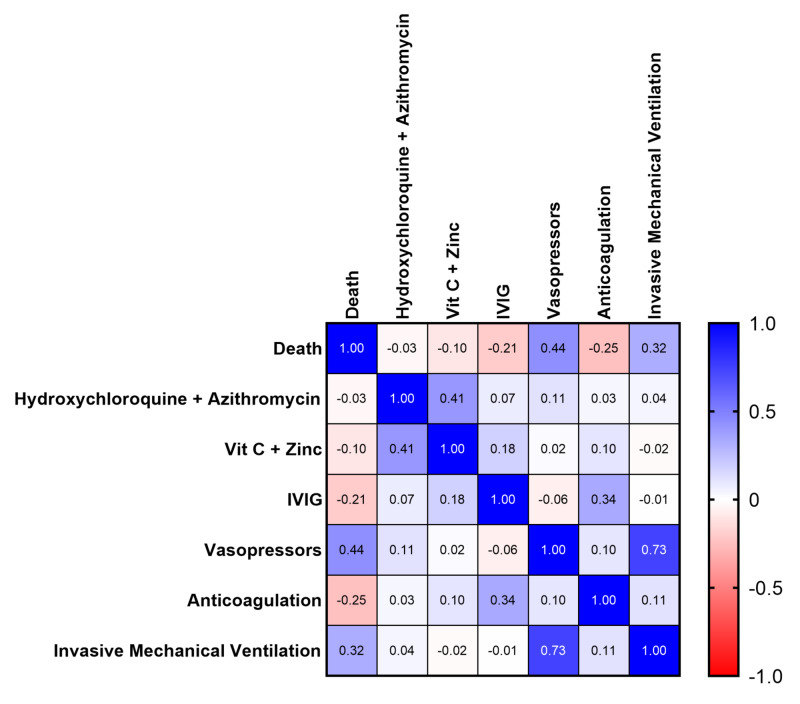
Correlation of Individual Medical Treatments to Likelihood of Death Denoted by Pearson r Coefficient IVIG: intravenous immunoglobin

Multiple linear regression analysis of each individual clinical treatment yielded multiple statistically significant results (Figure 3). Both treatment with hydroxychloroquine and azithromycin and vitamin C plus zinc were associated with no impact on overall survival (p=0.73; p=0.31), whereas both intravenous immunoglobin (IVIG) and anticoagulation were associated with an increased likelihood of survival, though low sample size should be noted (p=0.037; p=0.012). Overall all-cause mortality was 43.9% in patients receiving anticoagulation (18/41) and 25% in patients receiving IVIG (2/8). Various anticoagulation was used including heparin (24/41, 58.5%), enoxaprin (10/41, 24.2%), combination of heparin plus enoxaprin (4/41, 9.8%), or heparin plus tissue plasminogen activator (TPA; 2/41, 4.9%) and was unknown for one patient (1/41, 2.4%). As expected, use of vasopressor support and invasive mechanical ventilation were associated with a decreased likelihood of survival (p<0.001, p=0.001). 

**Figure 4:**
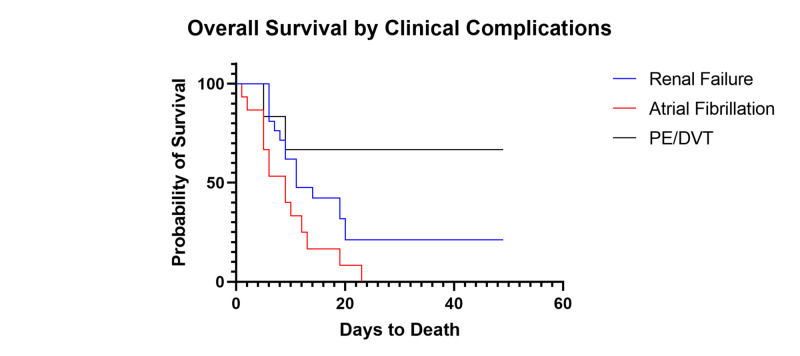
Survival of Patients Based on Clinical Complications PE: pulmonary embolism; DVT: deep vein thrombosis

We then analyzed patients based on new onset complications during their intensive care admission. Fifteen patients experienced new onset atrial fibrillation (15/102, 14.7%) without pre-existing cardiovascular disease, with 14 ultimately expiring (14/15, 93.3%). Of these patients, 13 were taking hydroxychloroquine and azithromycin at the onset of atrial fibrillation (13/15, 86.7%) and had no known cardiac history. We also report that 19 of these patients had new onset kidney failure requiring renal replacement therapy (19/102, 18.6%), with 12 deceased (12/19, 63.2%). Six patients had confirmed venous thrombotic events (pulmonary embolism or deep vein thrombosis; 6/102, 5.9%). Survival curve analysis showed significant differences in median OS based on presence of each individual complication (p=0.024, Figure 3). Median OS for was shorter for patients with atrial fibrillation versus those with renal failure (median OS 9 days vs. 11 days, respectively; Figure 4).

Impact of healthcare insurance on survival

**Figure 5:**
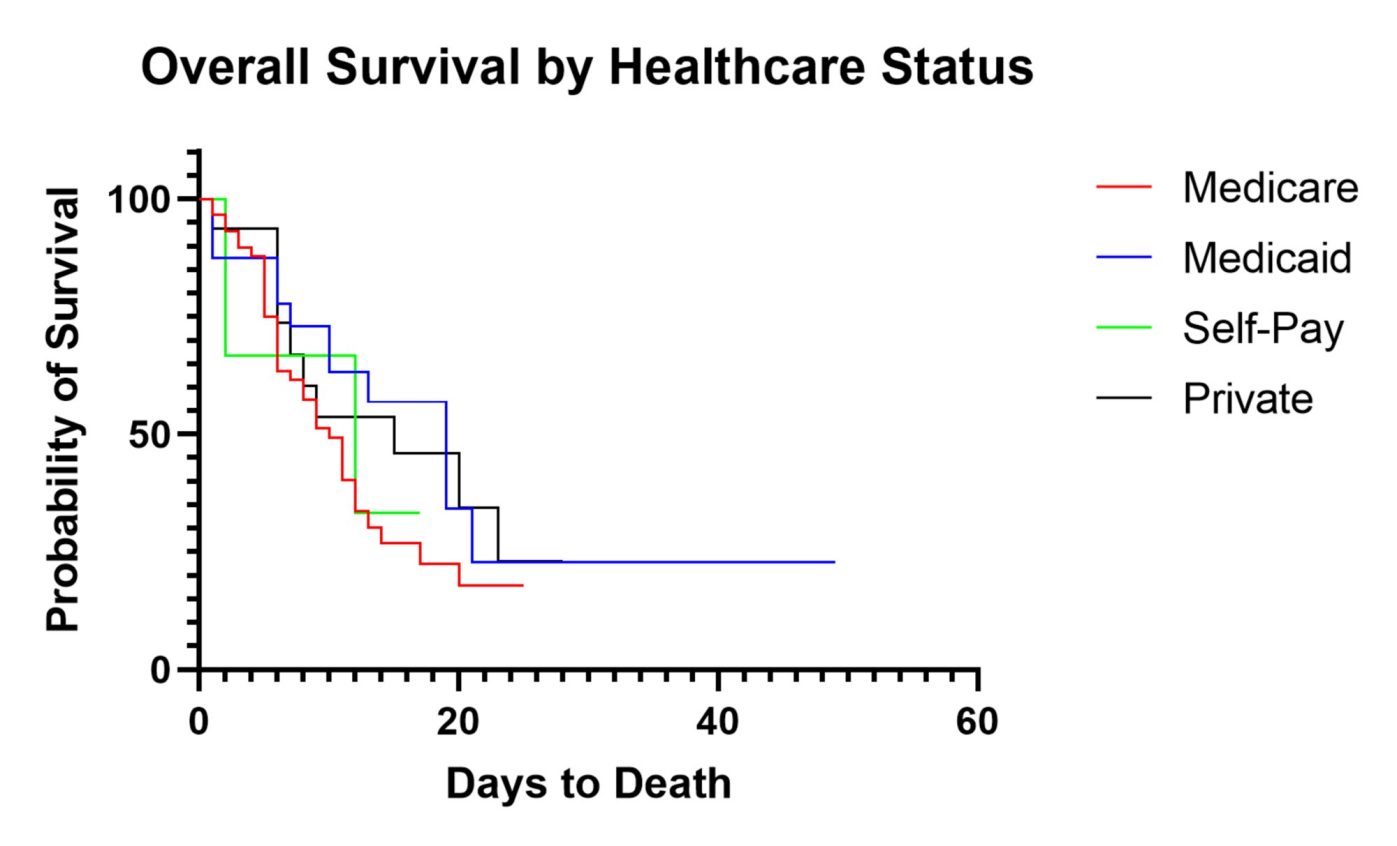
Survival of Critically Ill Patients Based on Insurance Status

We then investigated the impact of healthcare insurance status of the patient on overall survival. Patients were divided by insurance, of whom 83 (81.4%) were on either Medicare or Medicaid, which is consistent with our standard patient population. Further stratification showed 59 patients receiving Medicare (59/102, 57.8%) compared to 24 receiving Medicaid (24/102, 23.5%). Statistical analysis showed no significant difference in survival based on insurance status (p=0.38, Figure 5) with median overall survival of 10, 19, 12, and 15 days for Medicare, Medicaid, self-pay, and private insurance, respectively. 

Individual comorbidity impact on survival

**Figure 6:**
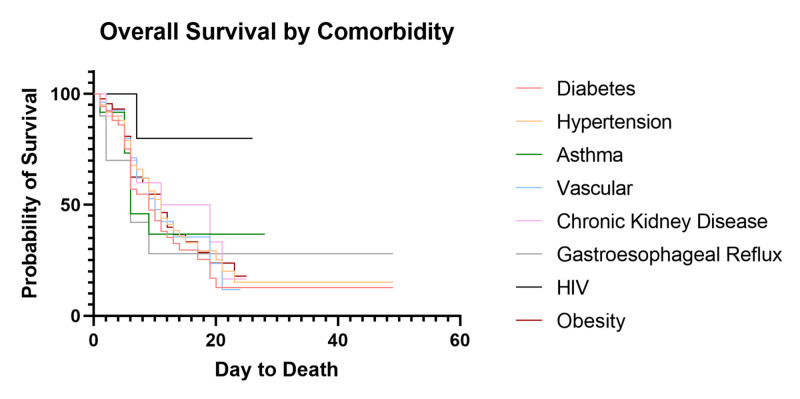
Survival of Critically Ill Patients Based on Individual Comorbidities HIV: human immunodeficiency virus

**Figure 7:**
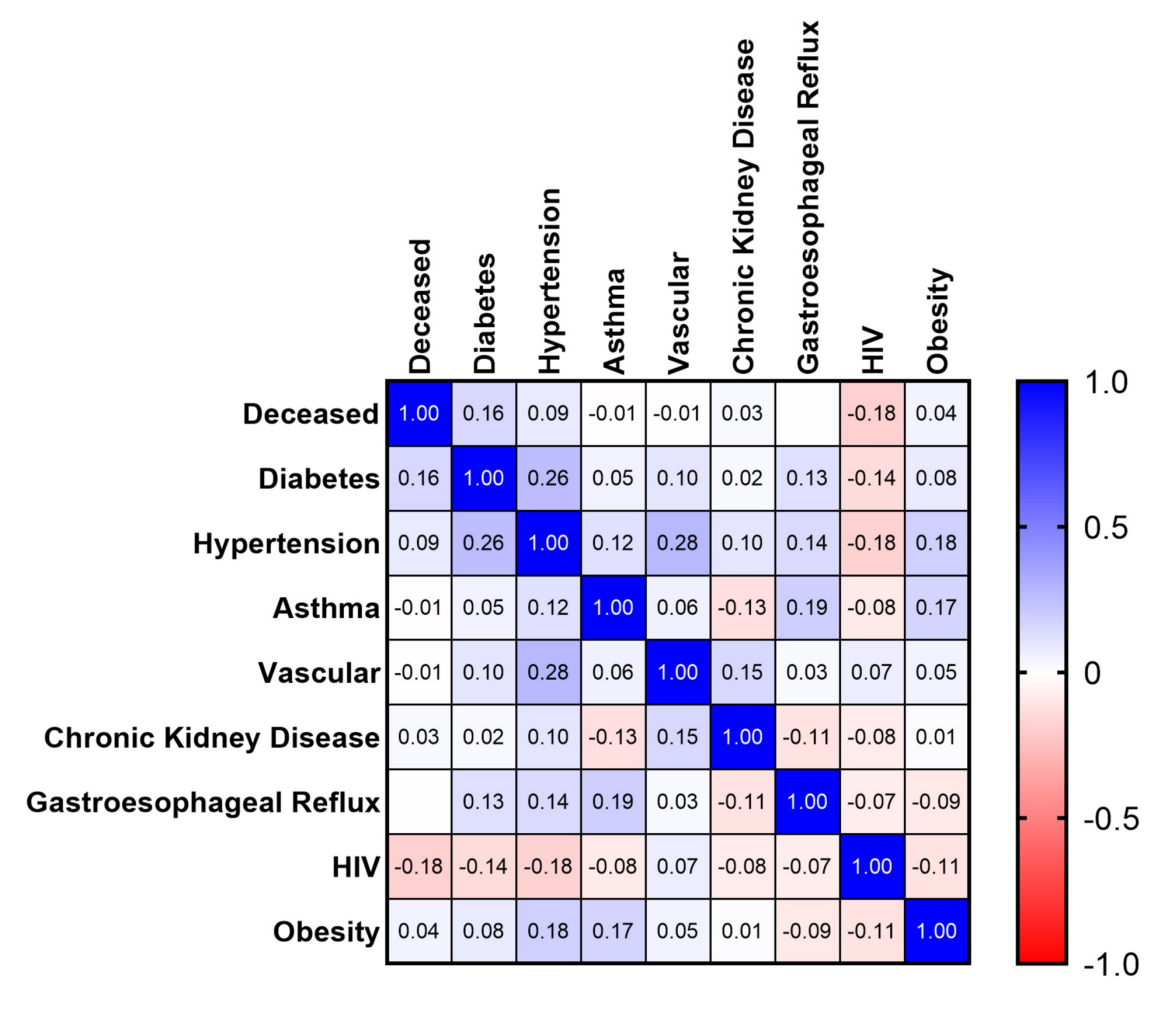
Correlation of Individual Comorbidities to Death Denoted by Pearson r Coefficient HIV: human immunodeficiency virus

We quantified prevalence of specific comorbidities, including obesity, diabetes, hypertension, asthma, vascular events, chronic kidney disease, gastroesophageal reflux disease, and human immunodeficiency virus (summarized in Table 1). Our population has similar rates of individual medical comorbidities to published populations [4-6], with 94.1% (96/102) of critically ill patients having one or more documented comorbidity and 75.5% (77/102) with two or more. Survival curve statistics for individual medical comorbidities were compared (Figure 6). When compared against themselves, there was no significant difference in median OS for a single comorbidity (p=0.62). We then aimed to see if an individual comorbidity had an impact on likelihood of death in these patients. Multivariate correlation analysis yielded no statistically significant results when comparing individual comorbidities to death (Figure 7) but showed a trend towards diabetes modestly decreasing survival (Pearson r=0.16; p=0.12). Deceased patients were then isolated and a multiple linear regression analysis was performed to compare each comorbidity to the overall days to death which yielded no statistically significant results. 

**Figure 8:**
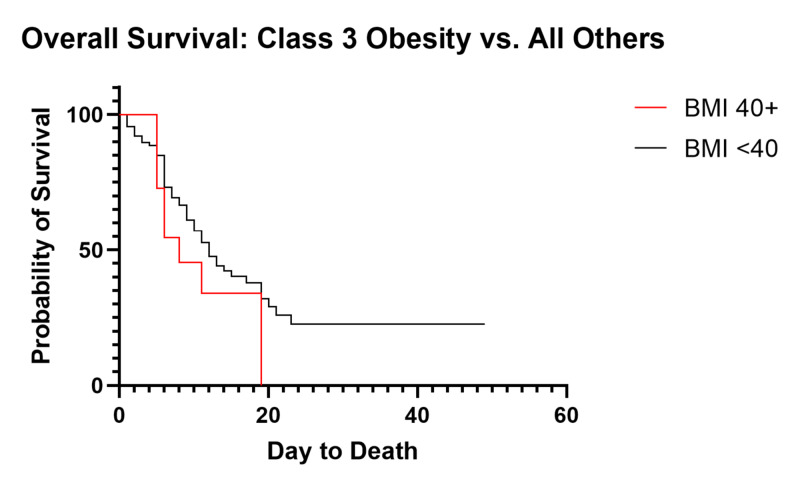
Overall Survival of Class 3 Obesity vs. All Others BMI: body mass index

Obesity has been suggested as a significant contributing factor to the progression of COVID-19 [12]. When analyzed separately, we show no significant difference in overall survival based on normal/overweight vs. obese (p=0.70, OS 12 vs. 11 days). As obesity can be stratified into subsets and anecdotal experience suggested an increased mortality with increased body mass index (BMI), we performed further analysis of OS within these subgroups. BMI can be stratified according to the Center for Disease Control and Prevention recommendations, separating obese into Class 1 (BMI 30-34.9), Class 2 (35-39.9) and Class 3 (40+) [11]. Underweight was defined as BMI <18.5, normal weight as 18.5-24.9, and overweight as 25-29.9. Survival curve analysis showed no difference in median OS between subsets of BMI (p=0.88), with median OS of 14 days, 10, 12, 13, and 8 for BMIs of normal, overweight, Class 1 obesity, Class 2 obesity, and Class 3 obesity, respectively, and median OS was undefined for underweight patients. Despite not being statistically significant, we see a trend towards increased mortality of 72.7% in Class 3 obesity (8/11) compared to mortality of 40.0% (2/5) in underweight patients, 55.0% (11/20) in normal weight, 54.8% (14/31) in overweight, 57.9% (11/19) in Class 1 obesity, and 56.3% (9/16) in Class 2 obesity. After separating Class 3 obesity and comparing to all others, we observe this trend (p=.19, Figure 8).

Total comorbidity impact on survival

**Figure 9:**
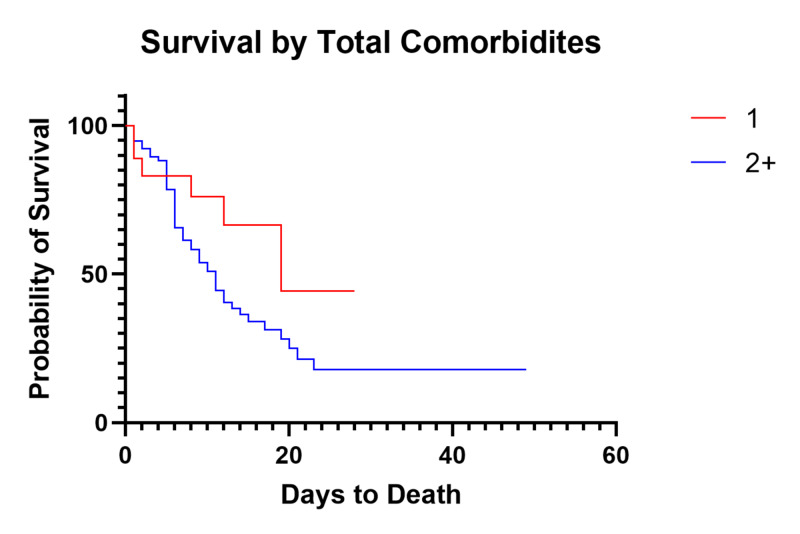
Survival of Critically Ill Patients Based on Total Comorbidities

Comparison between patients with a singular comorbidity (n=18) to those with two or more (n=77) showed a strong trend towards decreased survival (p=.16, Figure 9). Patients with no comorbidities were excluded due to low sample size. Median OS for patients with one comorbidity was 19 days, whereas median OS for patients with two or more comorbidities decreased to 11 days. 

The median Charlson Comorbidity Index (CCI) for the entire cohort was 3.389 (60% 1-year mortality) [13]. When compared between ethnicities, analysis showed no difference in CCI between groups (p=0.97). CCI averages of 3.23 vs. 3.67 vs. 1.80 were calculated for currently admitted, deceased, and discharged patients, respectively. Comparison between deceased vs. discharged patients shows a statistically significant decrease in CCI in discharged patients (3.67 vs. 1.80, p=0.024). 

## Discussion

Preliminary data across multiple states has shown a disproportionate number of minority patients being impacted by the COVID-19 pandemic [8-10]. Our findings support and extend these prior reports with 82.4% of our critically ill patients identified as African American or Hispanic. Further, we also observed a high prevalence of comorbidities in our cohort, which is likely a reflection of the social determinants of health (i.e. low income) in the population we serve. Here we present a comprehensive analysis of critically ill COVID-19 patients at our community safety-net hospital in Brooklyn, New York, with the most diverse patient population reported. 

We began by analyzing outcomes of patients based on treatment modalities. Prior work has suggested the efficacy of combination hydroxychloroquine and azithromycin, which has been heavily utilized at our institution, but we show no association with mortality or median OS (p=0.73), consistent with recently reported data [14-17]. It should be noted that the incidence of new-onset atrial fibrillation and renal failure were elevated in our patient population (15/102, 14.7%; 19/102, 18.6%, respectively), and atrial fibrillation was associated with hydroxychloroquine and azithromycin use in 86.7% of cases. Presence of new-onset atrial fibrillation was also associated with a poor prognosis, showing a decrease in median OS from 11 days to nine days and an overall mortality of 93.3%. This increased incidence of atrial fibrillation and the lack of impact on overall mortality and median overall survival suggests that the use of hydroxychloroquine and azithromycin must be carefully weighed against each individual patient’s risk factors and pre-existing comorbidities. While used in a minority of patients, intravenous immunoglobin and anticoagulation were both associated with an increased median OS. We suggest that the efficacy of anticoagulation may correlate to the increased incidence of thrombotic events seen in COVID-19 patients, which have the potential to lead to major sequelae, including organ failure and potentially death [18-20]. While these events themselves were relatively rare in our patients (5.9% overall), 40% of these patients ultimately succumbed to COVID-19. 

When dividing the patients based on ethnicity or by insurance status, analysis of both total mortality and median overall survival was not statistically significant. This was an unexpected finding, but we propose various reasons for these results. First, our population is disproportionately diverse and our comparator group of Caucasian patients had a small sample size (n=7). Second, ethnicity and healthcare status are not mutually exclusive from other poor prognostic indicators, mainly incidence of pre-existing comorbidities, and may in fact contribute to their presence. A lack of access to care among these patients is a significant underlying factor in the presence of comorbidities, which can ultimately lead to poor outcomes in COVID-19. While ethnicity and insurance status may not directly impact overall mortality or median overall survival, these factors must be considered on a larger scale as having an indirect impact.

Although there has been significant speculation that individual medical comorbidities may impact the outcomes of COVID-19 patients, we have been unable to identify any significant individual factor. We do note a trend towards decreased survival in patients with diabetes (p=0.12), but no individual factor showed a statistically significant impact on overall mortality or median OS. More likely, we propose that the overall health status of each individual patient based on the number of medical comorbidities plays a significant role in the progression and severity of COVID-19. This is evidenced by the strong trend showing a decrease in median OS of critically ill patients from 19 to 11 days with two or more pre-existing comorbidities (p=0.07) and an increased mortality of 63.6% (49/77) compared to 33.3% (6/18) in patients with a single comorbidity. This is consistent with our findings showing a strong trend towards lower CCI in discharged patients when compared to currently admitted and deceased patients. Obesity has also been suggested as a risk factor. Obesity as a whole did not show any association with overall survival or mortality, but when separating out Class 3 obesity, we note a trend towards increased mortality and decreased median OS. We suggest that this becomes a mechanical problem during severe COVID-19 acute respiratory distress syndrome (ARDS) due to increased intra-abdominal pressure, negative transpulmonary pressure, closure of dependent airways producing shunting and contributing to severe oxygenation failure, high positive end-expiratory pressure (PEEP), and hemodynamic instability coupled with the inability to overcome the weight of the chest wall [21,22]. 

There are multiple limitations of this study. First, we present statistics and outcomes from a single community hospital in Brooklyn, New York. Second, because the data was extracted from the electronic health record, there is the possibility that data was not documented in the system as it would be in the physical chart. Third, it was also assumed that discharged patients were alive at the cutoff date of April 20, 2020. Fourth, a significant portion of our critically ill patients remained admitted under the intensive care service (31/102, 30.4%) at our cutoff point. Fifth, patients with DNR orders were included in the study and did not undergo aggressive intervention. And lastly, we must consider the rapid nature of the changing understanding and treatment of COVID-19. Our management of these patients was based on the medical consensus at the time and continues to evolve with increased reporting of data and outcomes.

## Conclusions

Despite the fact that we show no significant differences in mortality based on ethnicity, insurance status, or individual medical comorbidities, we show a high overall mortality. There is also a trend towards increased overall mortality in Class 3 obesity, which should be further investigated. We suggest that these findings may be attributed to both socioeconomic factors and an increased incidence of total medical comorbidities in our patient population. Many of these variables are not mutually exclusive, with most of our patients being from a diverse population with limited access to medical care and multiple comorbidities. We also report no association between overall survival and hydroxychloroquine in combination with azithromycin, but increased overall survival was associated with patients that received supplemental anticoagulation or intravenous immunoglobin.
